# Assessment of clinical and biological characteristics by pathological subtypes of lung adenocarcinoma

**DOI:** 10.1007/s11748-026-02287-7

**Published:** 2026-03-19

**Authors:** Kohei Abe, Jun Suzuki, Hikaru Watanabe, Satoshi Takamori, Tetsuro Uchida, Satoshi Shiono

**Affiliations:** Department of Surgery II, Faculty of Medicine, Yamagata University, 2-2-2 Iida-Nishi, Yamagata City, Yamagata 990-9585 Japan

**Keywords:** Adenocarcinoma, Epidermal growth factor receptor, Pathological subtypes, Programmed death-ligand 1

## Abstract

**Background:**

Lung adenocarcinoma is classified into subtypes based on pathological features; however, the clinical and biological characteristics of each subtype, including driver mutations and programmed death-ligand 1 (PD-L1) expression, remain unclear. We aimed to clarify these characteristics.

**Methods:**

We retrospectively analyzed 1412 cases of stage I–III lung adenocarcinoma that underwent complete resection between 2004 and 2023. Clinical and biological characteristics were compared by predominant subtypes.

**Results:**

Among the 1412 cases, the predominant subtypes were papillary (n = 686, 48.6%), lepidic (n = 317, 22.5%), acinar (n = 234, 16.6%), solid (n = 166, 11.8%), and micropapillary (n = 9, 0.6%). The lepidic subtype was more common in females (58.0% vs. 43.5%; *p* < 0.001), had a lower smoking rate (47.3% vs. 60.9%; *p* < 0.001), smaller invasive size (8 mm vs. 18 mm; *p* < 0.001), higher frequency of epidermal growth factor receptor (EGFR) mutation (63.4% vs. 45.2%; *p* < 0.001), and lower PD-L1 expression (15.5% vs. 45.2%; *p* < 0.001). The solid subtype was more prevalent in males (76.5% vs. 50.2%) and smokers (83.1% vs. 54.5%), with a larger invasive size (20.1 mm vs. 15.0 mm), fewer EGFR mutations (12.6% vs. 54.2%; *p* < 0.001), and higher PD-L1 expression (66.6% vs. 35.2%; *p* < 0.001). The 5-year Recurrence-free survival rates for the lepidic, acinar, papillary, and solid subtypes were 88.2, 72.9, 65.1, and 58.2%, respectively (*p* < 0.001).

**Conclusion:**

Lung adenocarcinoma subtypes present distinct clinical and pathological profiles, which may affect treatment strategies and prognostic evaluation.

**Supplementary Information:**

The online version contains supplementary material available at 10.1007/s11748-026-02287-7.

## Background

Lung cancer remains the malignancy with the highest incidence and mortality rates worldwide. According to the global statistics for 2022, almost 2.5 million new lung cancer cases were diagnosed, with an age-standardized incidence rate of 23.6 per 100,000 individuals, accounting for about 18.7% of all cancer-related deaths [1]. Lung adenocarcinoma is the most common subtype of non-small cell lung cancer (NSCLC), accounting for over 40% NSCLCs. Its prevalence has steadily increased in recent years especially among non-smoking women. Additionally, complete resection is possible in the early stage of adenocarcinoma cases [2].

Adenocarcinoma is histologically classified into lepidic, acinar, papillary, solid, and micropapillary types [3]. Distinct clinical characteristics have been observed among subtypes. Particularly, the lepidic type is often observed in patients with no history of smoking [4]. A previous study reported that the 5-year overall survival (OS) rates after surgery for stage I–III lung adenocarcinoma were 70.9, 59.0, 41.0, and 40.5% for lepidic, acinar, solid, and papillary subtypes, respectively [5]. However, the underlying mechanisms of its development remain poorly understood [4, 6].

Recently, driver gene mutations and programmed death-ligand 1 (PD-L1) expression have garnered attention for their critical roles in guiding treatment decisions; adenocarcinoma with driver gene mutations could respond well to molecular targeted therapies. Additionally, the associations of different adenocarcinoma subtypes and their response to molecular targeted therapies have also become a point of interest [7, 8]. Biological characteristics and survival after surgery could be different among subtypes. Additionally, carcinogenesis and related clinical factors have not been clarified [9]. Different kinds of carcinogens could be associated with the different subtypes of adenocarcinoma. Given the different characteristics of each adenocarcinoma subtype, subtype-specific treatment approaches may be considered.

Based on these considerations, we aimed to evaluate the biological and clinical characteristics of each histological subtype of lung adenocarcinoma, with the potential to contribute to elucidating the developmental pathways of the disease and to the development of effective therapeutic strategies.

## Patients and methods

### Approval by institutional review board

This study was conducted in accordance with the principles outlined in the Declaration of Helsinki. It received approval from the institutional ethics committees, which also granted a waiver of informed consent (approval no. 2025-6) because all patient data remained anonymous.

### Study design

In this retrospective study, we included patients who underwent lung cancer surgery between January 2004 and December 2023 at two institutions in Yamagata Prefecture. We established a prospectively collected database including the following data: (i) preoperative patient demographics, (ii) tumor markers, (iii) the result of radiological findings, including chest computed tomography (CT) and positron emission tomography/CT, (iv) stage, (v) pathological findings, (vi) PD-L1 expression, (vii) driver mutation, (viii) recurrence pattern, and (ix) the day of recurrence and outcome. As the lung cancer tumor, node, and metastasis (TNM) staging system is revised approximately every decade, the classification transitioned from the previous edition to the 8th edition over the course of this study. For patients who underwent surgery before 2017, their TNM stage was reassessed according to the 8th edition criteria [10].

### Patient selection

During the study period, a total of 2951 patients underwent complete surgical resection for NSCLC. Among them, 2180 (73.9%) cases were adenocarcinoma. Cases with non-curative intent or stage IV disease were excluded. In addition, cases of lung cancer, those with missing data on the predominant pathological subtype, or those with incomplete data required for analysis were also excluded. The relationship between the biological and clinical characteristics and postoperative outcomes of each subtype were analyzed for the included cases (Supplementary Fig. 1).

### Pathological diagnosis

Pathologic stage was determined following the TNM classification of malignant tumors of the Union for International Cancer Control, 8th edition. The pathological diagnosis was made according to the World Health Organization (WHO) classification The diagnosis was performed by two pathologists at the institutions. The adenocarcinoma subtypes are explicitly defined in the 2015 revision of the WHO classification. Cases diagnosed prior to this update were reassessed based on a review of their pathological findings and collected database. Adenocarcinoma subtypes exhibit distinct histopathological features. Lepidic predominant tumors grow along alveolar structures with minimal stromal invasion and mild to moderate atypia. Acinar predominant tumors form well-defined glandular structures composed of cuboidal to columnar cells. Papillary predominant tumors have fibrovascular cores with crowded, stratified nuclei. Solid predominant tumors do not form glands, display sheet-like proliferation with high-grade atypia, and often have necrosis. Micropapillary predominant tumors consist of small papillary tufts without fibrovascular cores, frequently floating in alveolar spaces, and are strongly associated with lymphatic invasion (Supplementary Fig. 2) [3].

### Examination of driver mutations and PD-L1

Due to the long observation period and advancements in testing technology, various methods were used to examine EGFR mutation status. The polymerase chain reaction (PCR)–Invader method (BML Co., Tokyo, Japan), direct sequencing (SRL Co., Tokyo, Japan), and the peptide nucleic acid–locked nucleic acid PCR clamp method (LSI Medience Co., Tokyo, Japan) was employed. Recently, panel tests such as Oncomine Dx (Thermo Fisher Scientific, California, USA) and Amoy Diagnostics (Amoy Diagnostics Co., Fujian Province, China) have been utilized.

PD-L1 immunohistochemical staining was conducted on 4-μm-thick formalin-fixed, paraffin-embedded tissue sections using the Dako PD-L1 immunohistochemistry 22C3 pharmDx kit (Dako/Agilent, Santa Clara, USA) on the automated Dako Link 48 platform, following the manufacturer’s instructions. PD-L1 expression was assessed by calculating the tumor proportion score (TPS), representing the percentage of viable tumor cells (minimum of 100 cells) showing either full or partial membrane staining. The TPS results were categorized into three groups: negative (TPS < 1%), low expression (TPS between 1 and 49%), and high expression (TPS ≥ 50%).

### Postoperative surveillance

For the first 5 years following surgery, postoperative follow-up was conducted every 6 months and consisted of physical examinations, chest X-rays or CT scans, and blood biochemical tests. After this period, annual chest CT scans were performed up to 10 years post-surgery.

### Statistical analyses

Each subtype was compared against all other subtypes to assess distinct clinicopathological characteristics. Categorical variables were analyzed using chi-squared test or Fisher’s exact test, while continuous variables were compared using the Wilcoxon rank-sum test. Kaplan–Meier survival curves were constructed to estimate Recurrence-free survival (RFS), and differences between pathological subtypes were assessed using the log-rank test. The median follow-up duration was estimated using the reverse Kaplan–Meier method. RFS was defined as the time from the date of surgery to the date of death from any cause, the last follow-up or recurrence. OS was defined as the time from the date of surgery to death from any cause or the last follow-up. Patients who were alive or lost to follow-up at the last contact were censored. To further evaluate the prognostic impact of each subtype, Cox proportional hazards regression analyses were performed, and hazard ratios (HRs) with 95% confidence intervals (CIs) were calculated by comparing each subtype to all other subtypes combined.

For the analyses, we used EZR version 4.2.3 (Saitama Medical Center, Jichi Medical University, Saitama, Japan). Statistical significance was set at *p* < 0.05.

## Results

Of the 2951 patients who underwent complete surgical resection of NSCLC, we excluded cases with non-adenocarcinoma histology, incomplete resections and stage IV disease. A total of 2180 (73.8%) were diagnosed with lung adenocarcinoma. Of these, 1412 (64.8%) patients with resected stage I–III adenocarcinoma were included in the analysis (Supplementary Fig. 1). Predominant subtypes were papillary (n = 686, 48.6%), lepidic (n = 317, 22.5%), acinar (n = 234, 16.6%), solid (n = 166, 11.8%), and micropapillary (n = 9, 0.6%). The micropapillary subtype was excluded due to its small sample size.

We summarized the clinical characteristics of the four subtypes (Table 1). Significant differences in sex, smoking history, carcinoembryonic antigen (CEA) level, maximum standardized uptake value (SUVmax), forced expiratory volume in 1 s (FEV1.0%) predicted, pathological invasive tumor size, and pathological stage (*p* < 0.001) were observed. Regarding sex distribution, the proportion of female patients was > 50% in the lepidic subtype (58%), and < 50% in the solid subtype (23.5%). A similar trend was observed for smoking history: the proportion of patients with a smoking history was less than half in the lepidic subtype (47.3%), whereas it was markedly higher in the solid subtype (83.1%) (Table 1). Regarding the median CEA levels, the lepidic subtype had the lowest value (2.5 ng/mL), whereas the solid subtype exhibited the highest value (4.1 ng/mL). The median SUVmax was lowest in the lepidic subtype (1.8) and highest in the solid subtype (8.5). Early-stage disease was predominant in the lepidic subtype, with stage I accounting for 96.8% of cases. In contrast, the solid subtype exhibited the highest proportion of advanced-stage lung cancer. There was a significantly difference in pathological factors (v, ly, pl, and lymph node metastasis), among subtypes (Table 1). Solid subtype tends to have the most invasive pathological characteristics.

**Table 1:** The characteristics of each subtype*

Characteristics	Lepidic, n = 317	Papillary, n = 686	Acinar, n = 234	Solid, n = 166	*p*
Female	184 (58.0)	325 (47.4)	109 (46.6)	39 (23.5)	< 0.001
Age, year median	71 (65–78)	72 (66–77)	71 (64–77)	70 (62.3–76)	0.085
Smoking history +	150 (47.3)	384 (56.0)	137 (58.5)	138 (83.1)	< 0.001
Brinkmann index	0 (0–520)	178 (0–800)	200 (0–800)	740 (308–1000)	< 0.001
CEA, ng/ml	2.45 (1.7–3.7)	3.0 (1.9–5.0)	3.1 (2.1–5.0)	4.1 (2.5 − 7.0)	< 0.001
%FVC, %	106.3 (95.7–116.5)	105.8 (95.2–116.7)	107.1 (95.6–117.3)	105.6 (94.2–116.3)	0.680
FEV1.0%, %	76.2 (70.2–80.5)	74.4 (68.8 –79.7)	74.1 (67.9 –79.4)	71.4 (63.2–76.6)	< 0.001
Pure solid tumor	97 (30.6)	502 (73.2)	189 (80.8)	162 (97.6)	< 0.001
SUVmax	1.8 (1.3–2.6)	3.8 (2.0–6.4)	4.2 (2.2–7.6)	8.5 (4.6–12.7)	< 0.001
cN +	5 (1.6)	39 (5.7)	12 (5.1)	27 (16.3)	< 0.001
Invasive size, mm	8 (6 –11)	18 (13–25)	17 (11–25)	20 (15–28)	< 0.001
v +	7 (2.2)	87 (12.7)	26 (11.1)	46 (27.7)	< 0.001
ly +	9 (2.8)	63 (9.2)	27 (11.5)	26 (15.7)	< 0.001
pl +	6 (1.9)	123 (17.9)	64 (27.4)	52 (31.3)	< 0.001
pN +	8 (2.5)	116 (16.9)	30 (12.8)	27 (22.3)	< 0.001
pStage, I / II-III	307 (96.8) / 10 (3.1)	533 (77.7) / 153 (22.3)	195 (83.3) / 39 (16.7)	119 (71.7) / 47 (28.3)	< 0.001

FEV1.0%, Forced Expiratory Volume in 1 Second, percent predicted; CTR, Consolidation tumor ratio; SUVmax, Maximum Standardized Uptake Value; v, Vascular invasion; ly, Lymphatic invasion; pl, Pleural invasion; N, lymph node metastasis; pStage, Pathological Stage

^*^Data are presented as n (%) or median (interquartile range)

**Fig. 1:**
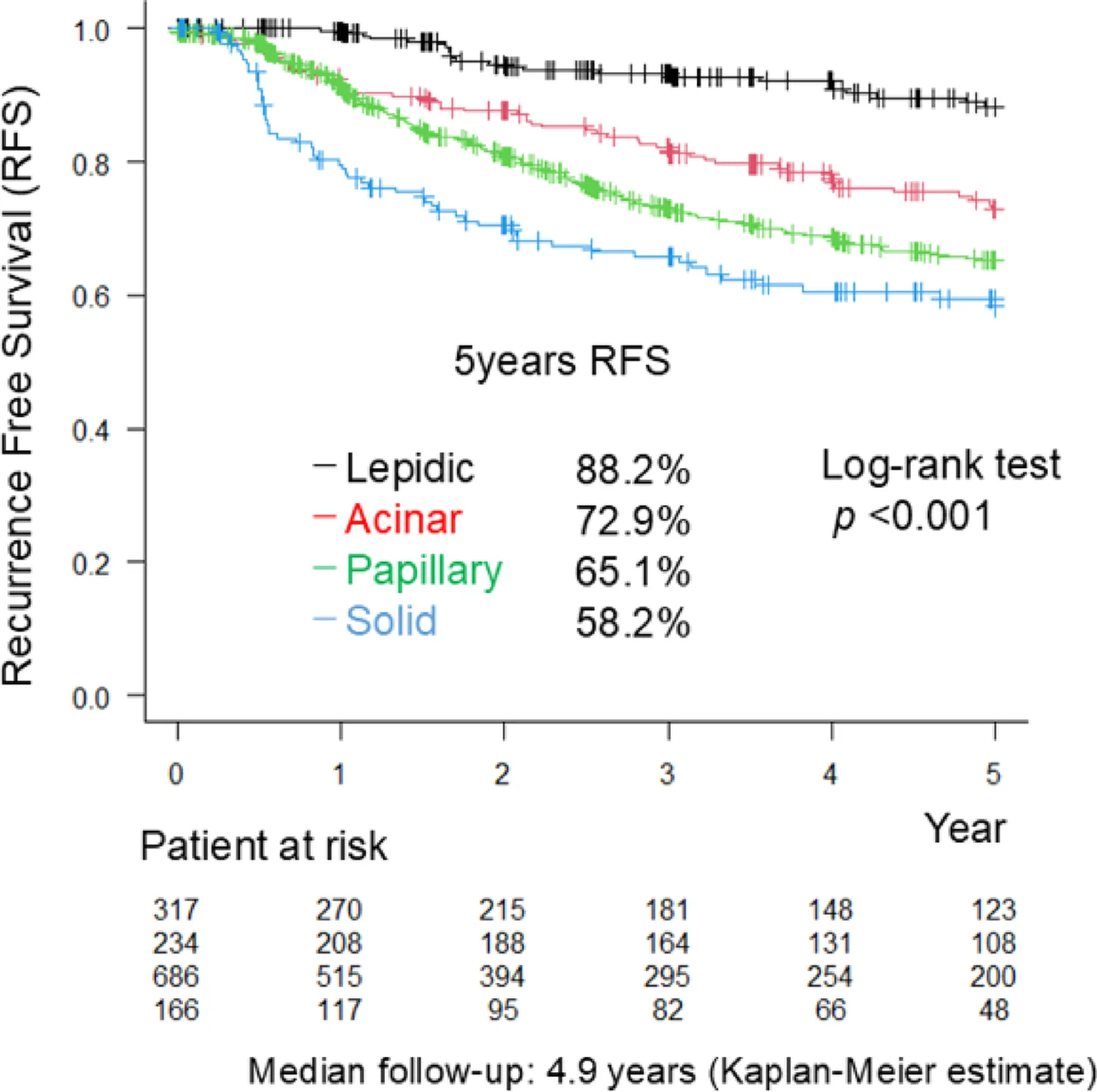
Recurrence free survival for each adenocarcinoma subtype. The p values were obtained using log-rank tests

RFS was significantly different among subtypes (*p* < 0.001) (Fig. 1). The 5-year RFS for the lepidic, acinar, papillary, and solid subtypes were 88.2, 72.9, 65.1, and 58.2%, respectively. Median follow-up time was 4.9 years. The HRs for RFS of each pathological subtype compared with all other subtypes were as follows: lepidic, 0.35 (95% CI 0.25–0.48; *p* < 0.001); acinar, 0.95 (95% CI 0.84–1.08; *p* = 0.439); papillary, 1.12 (95% CI 1.05–1.20; *p* < 0.001); and solid, 1.12 (95% CI 1.06–1.18; *p* < 0.001). We also observed significant differences among the histological subtypes in the pairwise log-rank test for RFS (Supplementary Table 1).

**Fig. 2 Recurrence free survival of stage I by subtypes:**
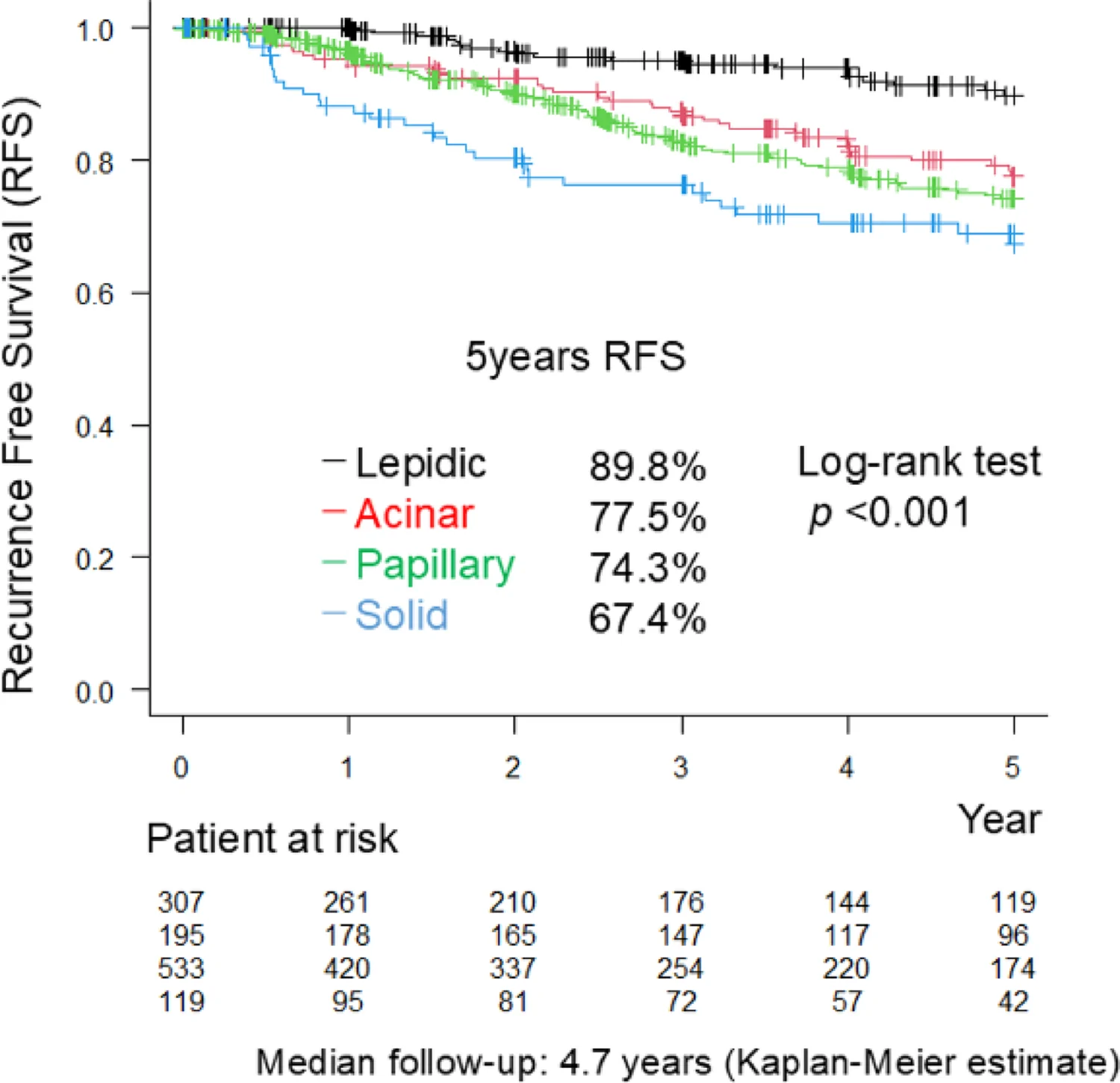
5

As there is a heterogeneity of the stages in this cohort, we carried out the same analysis for the stage I patients. Similar results were obtained even when limited to stage I disease (*p* < 0.001) (Fig. 2). The 5-year RFS of stage I for the lepidic, acinar, papillary, and solid subtypes were 89.8, 77.5, 74.3, and 67.4%, respectively. The HR for RFS of each pathological subtype compared with all other subtypes were as follows: lepidic, 0.44 (95% CI 0.31 – 0.63; *p* < 0.001); acinar, 0.98 (95% CI 0.83 – 1.14; *p* = 0.775); papillary, 1.10 (95% CI 1.01 –1.20; *p* = 0.025); and solid, 1.13 (95% CI 1.05 –1.21; *p* < 0.001). Even when the analysis was limited to stage I disease, differences in overall survival among the subtypes were observed (Supplementary Table 2). Kaplan–Meier curves of OS in both the Stage I–III and Stage I cohorts also revealed subtype-specific differences (Supplementary Fig. 3, 4) (Supplementary Table 3, 4).

**Fig. 3:**
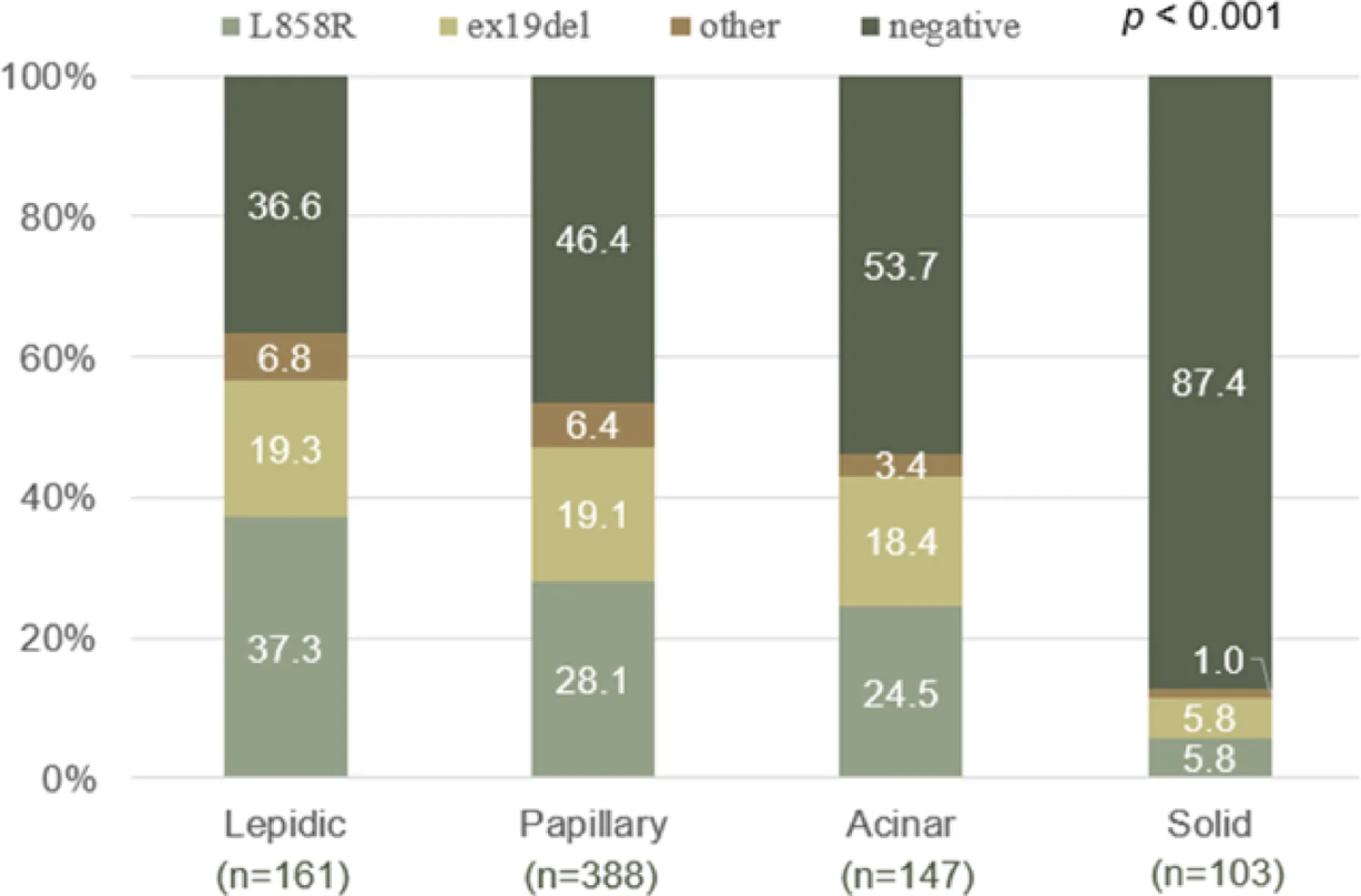
Association of pathological subtypes with EGFR mutations EGFR, epidermal growth factor receptor; ex19del, exon 19 deletion; L858R, exon21 L858R

Of the 1412 cases included in the study, driver mutation testing was available in 802 cases (56.8%). EGFR mutations were identified in 63.4% of cases in the lepidic subtype and in 11.6% in the solid subtype. The frequency of the L858R mutation was higher than that of exon 19 deletion in the non-solid subtypes; however, in the solid subtype, the two mutation types were observed at comparable frequencies (Fig. 3).

**Fig. 4:**
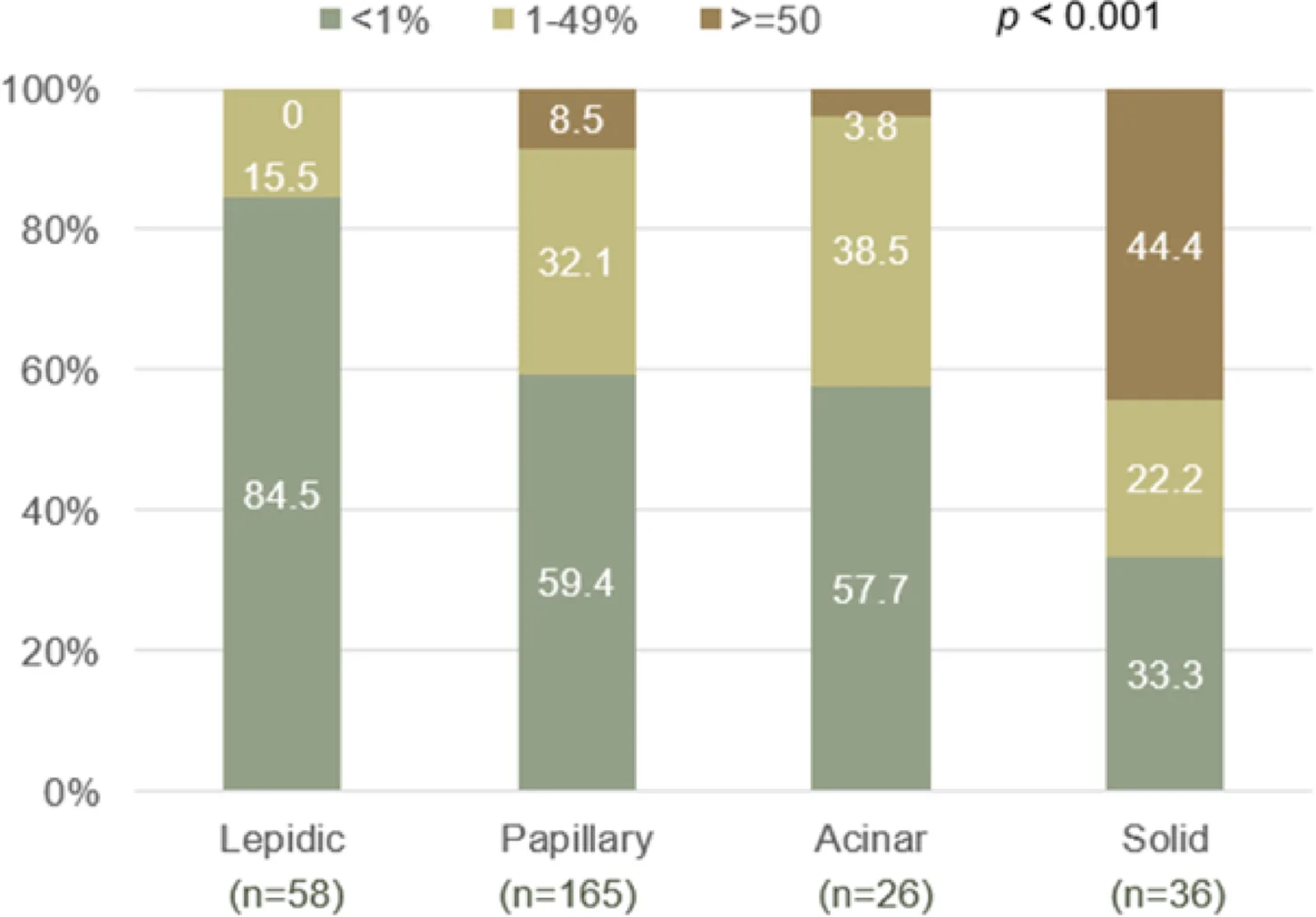
Association of pathological subtypes with PD-L1 expression PD-L1, programmed death-ligand 1

PD-L1 expression was assessed in 286 patients (20.3%). In the lepidic subtype, 84.5% of tumors showed low PD-L1 expression (< 1%), and no cases exhibited expression ≥ 50%. In contrast, PD-L1 expression was detected in 66.6% of solid subtype tumors, with high expression (≥ 50%) accounting for 44.4%. (Fig. 4). We additionally explored subtype-specific recurrence patterns, including analyses combining histological subtype with PD-L1 expression and EGFR mutation status; however, no statistically significant differences were identified.

## Discussion

Lung adenocarcinoma is the most common subtype of NSCLC [2]. Adenocarcinoma is classified according to the WHO [3], into several histological subtypes, some of which are associated with smoking; for instance, the lepidic subtype is more common in never-smokers, whereas the solid subtype is often observed in smokers [11]. Our study indicated that the lepidic subtype was more common in non-smoking females and was associated with smaller tumor size, lower CEA levels, frequent EGFR mutations, and low PD-L1 expression. These results suggest that it develops through a different pathway than smoking-related tumors. Recent reports show that PM2.5 exposure may cause EGFR mutations and contribute to lung cancer [12], suggesting that environmental pollution plays an important role [13]. Furthermore, another report suggested that genetic factors, including driver mutations, contribute to the carcinogenesis of early-onset lung adenocarcinoma [14]. These findings suggest that its carcinogenesis may differ depending on the histological subtype, with contributions from both environmental and genetic factors. Understanding these differences is crucial for clarifying the carcinogenesis. However, the relationships between the present findings and carcinogenesis remain hypothetical, and these results should be interpreted with caution given the retrospective and observational nature of this study.

We also observed differences among the non-lepidic subtypes in this study. The acinar and papillary subtypes showed intermediate findings, in contrast to the lepidic and solid subtypes, and generally shared similar clinical characteristics. The solid subtype exhibited distinct clinical and molecular characteristics. It was more common in male smokers [15], tended to present with larger tumors, and was associated with higher serum CEA levels. Most solid subtype cases did not harbor EGFR mutations but frequently showed high PD-L1 expression, suggesting a different underlying biology. In addition, the solid subtype often carried KRAS mutations [16]. These differences in developmental pathways may suggest the need for subtype-specific treatment strategies. [17].

Although several studies have reported relationships between adenocarcinoma subtypes and driver mutations or PD-L1 expression, many details remain unclear [18]. In this study, we present detailed data on their clinical and biological characteristics. It can be speculated that the solid subtype, which is more frequent in smokers and exhibits high PD-L1 expression, may arise because of smoking-induced upregulation of PD-L1. Although the solid subtype showed the lowest survival in our cohort, the expanding use of immune checkpoint inhibitors (ICIs), could improve survival outcomes for patients with the solid subtype. Therefore, our study, which reflects real-world survival data from a period when ICI use was limited, provides highly valuable information on the relationships among subtype, EGFR and PD-L1 status, and survival outcomes. Further research including a comprehensive evaluation of other driver mutations is required.

The prognosis of lung adenocarcinoma varies according to histological subtype. Significant differences were observed not only in OS but also in RFS, which reflects outcomes prior to the potential influence of post-recurrence systemic therapies guided by EGFR mutation status or PD-L1 expression. In this study, prognosis worsened in the order of lepidic, acinar, papillary, and solid subtypes. These RFS results were nearly identical even when the analysis was limited to Stage I. These results indicate that tumor aggressiveness could be significantly related with histological findings. The lepidic type usually has a better outcome. This may be because the tumor grows slowly and often responds well to EGFR inhibitors [19]. On the other hand, the solid type is more common in smokers and grows more aggressively. Patients with solid type lung adenocarcinomas often have comorbidities, which can lead to worse health results [20]. Additionally, due to the lower frequency of driver gene mutations, molecular targeted treatments are not used as much for the solid type tumors, worsening the prognosis. However, the growing use of ICIs gives hope for improved treatment for this subtype [15].

In the present study, a modest discrepancy was observed between recurrence-free survival and overall survival in the acinar and papillary subtypes; however, no differences were found in the prevalence of EGFR mutations or anaplastic lymphoma kinase (ALK) rearrangements (Supplementary Table 6), and detailed data on post-recurrence treatments were unavailable, although differences in the therapeutic effects of EGFR-TKIs after recurrence may have contributed to this finding.

Our study provides detailed clinicopathological and molecular data for each histological subtype of lung adenocarcinoma, with a median follow-up duration of 4.9 years. Because most previous reports assessed these factors separately or in relatively small cohorts, we summarized representative prior studies in a supplementary Table (Supplementary Table 7)[21-24].

Because treatment decisions in routine clinical practice are primarily guided by molecular profiling, including EGFR mutation status and PD-L1 expression, the direct clinical application of the present findings remains limited at this stage. Nevertheless, our results provide complementary insights into the biological heterogeneity underlying histological subtypes. We hypothesize that adenocarcinoma may display heterogeneous characteristics even within the same subtype, and further classification based on molecular features may help refine our understanding of tumor biology.

As noted above, the present study primarily provides descriptive data, and its direct application to routine clinical practice remains limited. However, our findings may generate hypotheses for future clinical application. For example, in patients without established biomarkers or PD-L1 expression, in whom treatment strategies at recurrence are often difficult to determine, those with subtypes associated with a higher risk of recurrence might benefit from the consideration of more intensive adjuvant therapy.

## Limitations

This study has some limitations. First, it was a retrospective analysis based on data from two local institutions, which may introduce selection bias and limit the generalizability of the findings. A large-scale, multicenter prospective study is necessary to validate and build upon these results. Second, although we classified tumors according to their predominant pathological subtype, adenocarcinomas often contain a mixture of subtypes. Analyzing tumors based on the proportional composition of each subtype may offer a more detailed understanding of their clinical and molecular characteristics. Third, although spread through air spaces is considered one of the prognostic factors, it could not be included in the analysis due to insufficient data. Fourth, in this study, the use of resected specimens allowed for the identification of subtypes; however, it is often difficult to determine subtypes from preoperative biopsy samples. This remains a challenge for developing non-surgical or preoperative treatment strategies based on subtype in the future. Fifth, PD-L1 expression and EGFR mutation status were not assessed in all cases; these tests were more frequently performed in advanced or recurrent disease, raising the possibility of selection bias. In addition, no adjustment for multiple comparisons was performed, which represents a limitation of the present study. Finally, although historical cases may significantly influence the interpretation of survival outcomes, these cases may represent the true biological characteristics of lung cancer. With the widespread adoption of TKIs and ICIs, the intrinsic characteristics of lung cancer may be increasingly difficult to observe. The present study may help to elucidate the intrinsic characteristics of lung cancer.

## Conclusion

Our findings show that the subtypes of lung adenocarcinoma are not only histological patterns but also biologically and clinically meaningful groups with distinct carcinogenetic pathways. Recognizing these differences is important for tailoring treatment. Our data may contribute to a better understanding of the biological heterogeneity of adenocarcinoma and may help inform the future development of subtype-specific treatment strategies.

## Supplementary Information

Below is the link to the electronic supplementary material.

Supplementary Material 1 Study schema

Supplementary Material 2 Pathological images for each subtype (a) Papillary predominant tumor with fibrovascular cores and stratifiednuclei (b) Lepidic predominant tumor growing along alveolar wallswith minimal stromal invasion (c) Acinar predominant tumor formingwell-defined glandular structures (d) Solid predominant tumor lackinggland formation, with sheet-like growth and marked atypia

Supplementary Material 3 Kaplan−Meier survival curves for each adenocarcinoma subtype

Supplementary Material 4 5-year overall survival of stage I by subtypes

Supplementary Material 5
