# Supplementary material for: Assessment of clinical and biological characteristics by pathological subtypes of lung adenocarcinoma
Source: Gen Thorac Cardiovasc Surg. 2026 Mar 19;74(9):871–9. doi: 10.1007/s11748-026-02287-7 (PMC13506621; doi:10.1007/s11748-026-02287-7)
Supplement: Supplementary file 1 [file 11748_2026_2287_MOESM5_ESM.docx]

Supplementary Table 1: Pairwise log-rank test for recurrence free survival among histological subtypes

| Comparison | *p* |
| --- | --- |
| Lepidic vs. Acinar | < 0.001 |
| Lepidic vs. Papillary | < 0.001 |
| Lepidic vs. Solid | < 0.001 |
| Acinar vs. Papillary | 0.133 |
| Acinar vs. Solid | < 0.001 |
| Papillary vs. Solid | < 0.001 |

Supplementary Table 2: Pairwise log-rank test for recurrence free survival of stage I among histological subtypes

| Comparison | *p* |
| --- | --- |
| Lepidic vs. Acinar | < 0.001 |
| Lepidic vs. Papillary | < 0.001 |
| Lepidic vs. Solid | < 0.001 |
| Acinar vs. Papillary | 0.810 |
| Acinar vs. Solid | < 0.001 |
| Papillary vs. Solid | < 0.001 |

Supplementary Table 3: Pairwise log-rank test for overall survival among histological subtypes

| Comparison | *p* |
| --- | --- |
| Lepidic vs. Acinar | 0.023 |
| Lepidic vs. Papillary | < 0.001 |
| Lepidic vs. Solid | < 0.001 |
| Acinar vs. Papillary | 0.011 |
| Acinar vs. Solid | 0.011 |
| Papillary vs. Solid | 0.409 |

Supplementary Table 4: Pairwise log-rank test for overall survival of stage I among histological subtypes

| Comparison | *p* |
| --- | --- |
| Lepidic vs. Acinar | 0.024 |
| Lepidic vs. Papillary | < 0.001 |
| Lepidic vs. Solid | < 0.001 |
| Acinar vs. Papillary | < 0.001 |
| Acinar vs. Solid | 0.012 |
| Papillary vs. Solid | 0.409 |

Supplementary Table 5: Surgical procedures by histological subtypes

| Characteristics | Lepidic, n = 317 | Papillary, n = 686 | Acinar, n = 234 | Solid, n = 166 | *p* |
| --- | --- | --- | --- | --- | --- |
| Surgical procedures |  |  |  |  | < 0.001 |
| Lobectomy | 190 (59.9) | 524 (76.4) | 185 (79.1) | 127 (76.5) |  |
| Segmentectomy | 98 (30.9) | 111 (16.2) | 26 (11.1) | 13 (7.8) |  |
| Wedge resection | 29 (9.1) | 51 (7.4) | 23 (9.8) | 26 (15.7) |  |

Supplementary Table 6: ALK mutation by histological subtypes

| Characteristics | Lepidic, n = 121 | Papillary, n = 262 | Acinar, n = 92 | Solid, n = 64 | *p* |
| --- | --- | --- | --- | --- | --- |
| ALK + | 1 (0.1) | 5 (1.9) | 4 (4.3) | 4 (6.1) | 0.100 |

ALK, anaplastic lymphoma kinase

Supplementary Table 7. A summary table of prior representative studies.

|  | Subtypes | PD-L1 | EGFR | Outcome |
| --- | --- | --- | --- | --- |
| Kojima K. 2022 | N=356 | 〇 | － | － |
| Russell PA . 2016 | N=575 | － | － | 〇 |
| Nakamura H. 2015 | N=320 | － | 〇 | 〇 |
| Zhen C. 2014 | N=206 | － | 〇 | － |
| Present data | N=1412 | 〇 | 〇 | 〇 |
